# Intimate partner violence is associated with poorer maternal mental health and breastfeeding practices in Bangladesh

**DOI:** 10.1093/heapol/czaa106

**Published:** 2020-11-09

**Authors:** Lan Mai Tran, Phuong Hong Nguyen, Ruchira Tabassum Naved, Purnima Menon

**Affiliations:** c1 Alive &Thrive, FHI360,18 Ly Thuong Kiet Street, Hanoi, Vietnam; c2 Poverty, Health and Nutrition Division, International Food Policy Research Institute, Washington, DC, USA; c3 Health System and Population Studies Division, ICDDR, GPO Box 128, Dhaka 1000, Bangladesh

**Keywords:** Intimate partner violence, determinants, mental health, breastfeeding, Bangladesh

## Abstract

Exposure to intimate partner violence (IPV) can have profound adverse consequences on maternal and child health. This study aimed to: (1) identify factors associated with IPV during pregnancy and postpartum in Bangladesh; and (2) assess the associations between IPV and maternal mental health and breastfeeding practices. We used data from a cross-sectional survey of 2000 mothers with children <6 months in four districts in Bangladesh. We applied multivariable logistic regression models to examine factors associated with IPV and structural equation modelling to assess the inter-relationships between IPV, maternal common mental disorders (CMD, measured by Self-reporting Questionnaire ≥7) and breastfeeding practices. Overall, 49.7% of mothers experienced violence during the last 12 months and 28% of mothers had high levels of CMD. Only 54% of women reported early initiation of breastfeeding and 64% reported exclusive breastfeeding. Women were more likely to experience IPV if living in food-insecure households, being of low socio-economic status, having low autonomy or experiencing inequality in education compared with husbands (OR ranged from 1.6 to 2.8). Women exposed to IPV were 2–2.3 times more likely to suffer from high levels of CMD and 28–34% less likely to breastfeed their babies exclusively. The indirect path (the indirect effects of IPV on breastfeeding through CMD) through maternal CMD accounted for 14% of the relationship between IPV on breastfeeding practice. In conclusion, IPV is pervasive in Bangladesh and is linked to increased risks of CMD and poor breastfeeding practices. Integrating effective interventions to mitigate IPV, along with routine maternal and child health services and involving men in counselling services, could help both to reduce exposure to IPV among women and to contribute to better health outcomes for women and children.

**KEY MESSAGES**

- Intimate partner violence (IPV) has profound adverse consequences on maternal and child health.
- Women with low autonomy, living in poor households, experiencing food insecurity and having inequality in education (compared with their spouses) were more likely to experience IPV.
- Women who experienced IPV were more likely to be depressed and less likely to practise exclusive breastfeeding.
- Health systems play a crucial role in a multisectoral response to violence against women. Integrating effective approaches to screen for and address IPV along with reproductive, maternal and child health services could be a double duty action to address the high prevalence of IPV, and improve maternal mental health and suboptimal breastfeeding practices.
- As one of the countries with the largest burden of IPV and also the highest prevalence of maternal and child undernutrition, integrating screening and support interventions into the health system and across other programmes in Bangladesh has the potential to reduce IPV, and thus also help achieve several Sustainable Development Goals related to empowering women, promoting gender equality and improving health and nutrition outcomes.

## Introduction

Intimate partner violence (IPV)—defined as physical, sexual and emotional abuse and controlling behaviours by an intimate partner—is a common form of violence against women. The lifetime prevalence of physical and/or sexual IPV against women is estimated at 30% globally, ranging from 23% in high-income countries to 38% in the low- and middle-income countries (LMICs) (WHO, 2013a). Bangladesh has one of the highest rates of IPV (Garcia-Moreno *et al.*, 2005), with two-thirds of ever-married women reporting at least one form of IPV during their lifetime and half reporting any form of IPV during the past 12 months (Bangladesh Bureau of Statistics, 2016). IPV during pregnancy and after childbirth is also common, with >30% of women experiencing physical IPV and 60% emotional IPV (Islam *et al.*, 2017b).

Drivers or triggers of IPV have been identified at multiple levels: individual, relationship, community and societal (WHO, 2012). Bangladesh has shared similar determinants of IPV to many other LMICs (Hindin *et al.*, 2008). At the individual level, women with low levels of education, who married as children, who are in employment and whose husbands have extramarital relationships were significantly more likely to experience IPV (Naved *et al.*, 2017b). Gambling and substance abuse, increasingly widespread, are more recent factors that are driving IPV (Naved *et al.*, 2017b). Men’s perceptions of being disadvantaged relative to women also contribute to IPV (Naved *et al.*, 2017b). At the household level, poverty and food insecurity are the key factors associated with IPV (VanderEnde *et al.*, 2015). Other household factors associated with IPV include women’s decision-making power, the quality of the marital relationship, the number of partners a man has, the number of children in the household (Naved *et al.*, 2017b) and inequality in education between women and their husband (Rapp *et al.*, 2012). In terms of community-level factors, inequitable community gender norms have been shown to increase the likelihood of IPV (Yount *et al.*, 2018). Risk factors for IPV during pregnancy are often similar to those for IPV in general (Jewkes, 2002; WHO, 2011). However, some other factors are likely to be more important during pregnancy in the Bangladeshi context, such as unwanted or unplanned pregnancies (Hindin *et al.*, 2008) and a history of abuse (Naved *et al.*, 2017b). No clear evidence exists about whether IPV itself increases or decreases during pregnancy (Jasinski, 2004).

IPV has profound adverse consequences on women’s mental health and childcaring behaviours. Women who had experienced IPV had higher levels of emotional distress (Ziaei *et al.*, 2016) and depressive symptoms (Devries *et al.*, 2013a, Parvin *et al.*, 2018), had lower mental health and social functioning scores (Dillon *et al.*, 2013) and were more likely to report suicidal ideation and attempts (Devries *et al.*, 2011, 2013a). In violent domestic environments, the quality of mothering and the ability of both parents to cope with children's needs are impaired (Kong and Lee, 2004; Victora *et al.*, 2010). Evidence on the influences of IPV on children's feedings are still scarce and inconsistent. A population-based study based on cross-sectional Demographic and Health Surveys from 51 LMICs showed that all three types of IPV were independently associated with a decreased likelihood of early initiation of breastfeeding (EIBF) (Caleyachetty *et al.*, 2019). In Bangladesh, women who experienced IPV after childbirth were also less likely to exclusively breastfed their infants (Islam *et al.*, 2017a). However, other studies did not find an association between IPV and early breastfeeding practices (Zureick-Brown *et al.*, 2015; Mezzavilla *et al.*, 2018).

Due to widespread violence against women and its importance for maternal and child health, research has increasingly focused on this topic, but studies have typically examined each aspect separately. A large body of literature has described the prevalence of IPV among women of reproductive age (Devries *et al.*, 2013b) and during pregnancy (Devries *et al.*, 2010; Halim *et al.*, 2018), and its associated risk factors (WHO, 2012). Another body of literature provides evidence on the influences of IPV on maternal physical and psychological health (Devries *et al.*, 2011, 2013a; WHO, 2013a). And yet another set of studies has focused on associations between IPV and childcare behaviours, including breastfeeding practices (Mezzavilla *et al.*, 2018; Caleyachetty *et al.*, 2019). Our study attempts to bring together an understanding of the linkages across these domains in the same context and using the same dataset. We aimed to: (1) examine the magnitude of different forms of IPV during pregnancy and postpartum periods; (2) identify factors associated with IPV; and (3) assess the influences of IPV on maternal mental health and breastfeeding practices.

## Methods

### Data sources

Data for this paper were obtained from a baseline household survey of women who had delivered a baby in the six months prior to the survey. Data were collected in 2015 as part of an evaluation to test the feasibility and impacts of integrating intensified maternal nutrition interventions into the existing maternal, newborn and child health programme platform in Bangladesh (Nguyen *et al.*, 2017b). The survey was carried out in 20 rural sub-districts (upazilas) from four districts (Mymensingh, Rangpur, Kurigram and Lalmonirhat). Within each sub-district, five unions and two villages within each union were randomly selected to yield a total of 200 villages (each had an average size of 250 households). Within each village, a household census was conducted to create a list of mothers with living infants <6 months of age. A total of 2000 mother–infant pairs were selected for the survey using systematic sampling, beginning with a random seed start point to yield the desired sample size per cluster.

Data were collected via face-to-face interviews using a structured questionnaire by researchers from Data Analysis and Technical Assistance Limited (DATA), an experienced and well-qualified survey firm in Bangladesh. Survey enumerators were trained by senior researchers using lectures, role play, mock interviews and practice in a classroom setting, and then field-tested the questionnaire; revisions were made to the questionnaire based on the results of field testing. The questionnaire was prepared initially in English and translated into Bangla, then back translated into English to double check for accuracy and consistency. All interviews were conducted one-to-one in a safe and private room at respondents' homes, and confidentiality was ensured for all women.

Ethical approval was obtained from the Institutional Review Boards of the BRAC University in Bangladesh and the International Food Policy Research Institute, USA. Written informed consent was obtained from all women ≥18 years of age. For women <18 years of age, we obtained their assent and the permission of their guardians, i.e. their parents or husbands, to participate in the study.

### Outcomes

The survey collected information on IPV experienced by recently delivered women using questions adapted from the World Health Organization (WHO) Multi-Country Study on Women’s Health and Domestic Violence (Garcia-Moreno *et al.*, 2005). The questions were validated for use in several countries including Bangladesh (Garcia-Moreno *et al.*, 2005), showing good internal consistency between each specific measurement (Cronbach’s alpha ranged from 0.75 to 0.90 for different types of IPV) (Islam *et al.*, 2017b).

The respondents were asked about their experience of a range of controlling behaviours and were asked to report whether they had experienced physical, emotional or sexual IPV by their intimate partner (Supplementary Table S1). Seven items were used to measure physical IPV: (1) pushing, shaking or throwing something at her; (2) slapping; (3) twisting her arm or pulling her hair; (4) punching or hitting with a fist or something harmful; (5) kicking or dragging or physically assaulting her; (6) choking or burning; (7) threatening or attacking with a knife, gun or any other weapon. Emotional IPV was measured by at least one affirmative response to questions asking whether or not the respondent's husband had insulted her or made her feel bad about herself; humiliated her in front of others; done things to scare or intimidate her on purpose; or threatened to hurt her or someone she cares about. A response was coded as sexual violence by an intimate partner if women reported having been physically forced to have sexual intercourse; having intercourse out of fear; or being forced to perform sexual acts that she found degrading or humiliating. The lifetime prevalence of partner violence is defined as the proportion of women who report having experienced one or more acts of violence at any point in their lives. Current prevalence is the proportion of women reporting that at least one act of domestic violence had taken place during the 12 months prior to the interview. Some women had been exposed to all three types of violence (emotional, physical and sexual).

Maternal common mental disorders (CMD), our outcome of interest, were measured using the 20-item Self-reporting Questionnaire (SRQ-20) (WHO, 1994). This tool was validated and adapted for screening mental disorders in developing countries (Harpham *et al.*, 2005) including Bangladesh (Khan and Flora, 2017). Each item is scored with 0 or 1, depending on responses related to CMD over the past 30 days. Cronbach’s alpha for the scale was 0.9 in our sample, indicating good internal consistency. The scores are added to generate an overall SRQ-20 scale, in which higher scores denote higher levels of maternal CMD (WHO, 1994). A cut-off of 7 was considered to ascertain maternal CMD, as recommended by some studies (Stewart *et al.*, 2008; Medhin *et al.*, 2010) including previous studies in Bangladesh (Khan *et al.*, 2008; Ziaei *et al.* 2016; Khan and Flora, 2017).

Breastfeeding practices were assessed using survey questions to construct standard WHO indicators (Akman *et al.*, 2008) for EIBF (defined as the proportion of children born in the last 24 months who were put to the breast within 1 h of birth) and exclusive breastfeeding (EBF) (defined as the proportion of infants 0–5 months of age who had been fed exclusively with only breast milk in the previous 24 h). Optimal breastfeeding practice was defined as children who had been breastfed immediately after birth and had been exclusively breastfed in the 24 h preceding the survey.

### Potential factors associated with IPV

**Figure 1:**
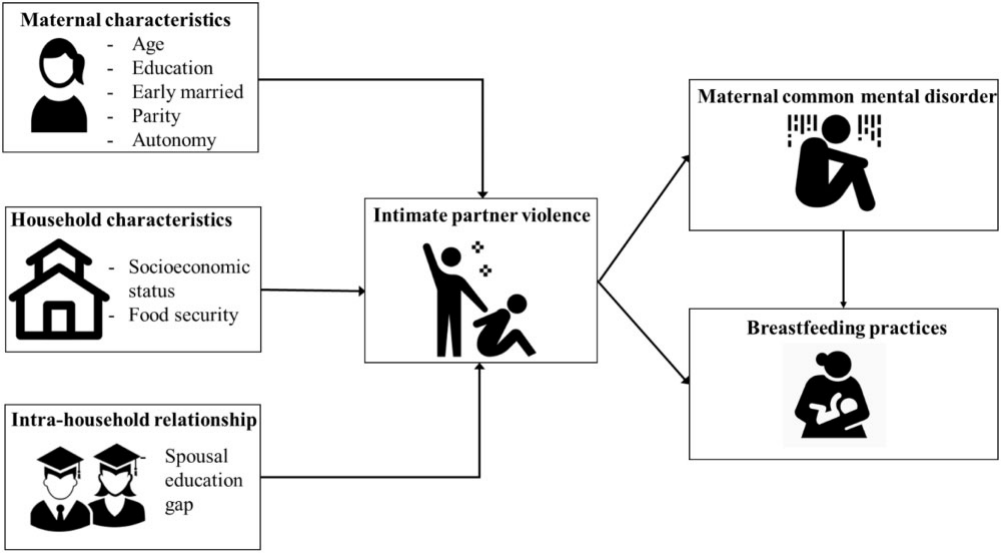
Conceptual framework for determinants of IPV and influences of IPV on maternal mental health and child feeding practices

We were not able to find any comprehensive frameworks for determinants of IPV or influences of IPV on maternal mental health and breastfeeding practices. Therefore, we developed a conceptual framework based on a literature review to guide our analyses (Figure 1), considering maternal, household levels and the potential influence of intra-household relationships. IPV may influence breastfeeding practices directly or indirectly through maternal CMD. Although we show directionality using arrows in Figure 1 for illustrative purposes, we acknowledge the interplay of factors presented in the framework, and the potential bidirectional nature of the links between them.

#### Maternal and child factors

Maternal characteristics that were examined include age, education (categorized as illiterate, elementary school, middle school and high school or higher) and parity. Age at first marriage was recorded, and we created a variable for early married (1, 0) using a cut-off of <19 years of age. Details on mode of delivery (caesarean section), child age and sex were used as controlled variables in the models of breastfeeding practices.

Women’s autonomy index was measured based on a set of 27 items that covered four different dimensions: women’s economic decision-making autonomy, familial healthcare and family planning decision-making autonomy, women’s freedom of movement autonomy and women’s attitudes towards gender. Details of these items were presented in Supplementary Table S2. For each of the questions in the first three dimensions, the responses were coded as 1 (respondent decided alone or jointly) or 0 (respondent did not make decision). The index of women’s attitudes towards gender dimension was measured by asking women whether they agreed or disagreed with statements on women’s roles. Each statement was given a score of 1 (agree) or 0 (disagree). The sums were used as an overall autonomy index (range: 2–27), and this index was divided into tertiles to obtain high, medium and low categories.

#### Household factors

Two key factors were considered at the household level: household socio-economic status (SES) and food security. An index for household SES was constructed using a principal components analysis of variables on housing conditions and asset holdings, and the first component derived from component scores was used to divide the SES score into tertiles (Vyas and Kumaranayake, 2006; Gwatkin *et al.*, 2007). Household food security was measured and calculated using FANTA/USAID’s Household Food Insecurity Access Scale (Coates *et al.*, 2007), which provides information related to the household’s experience of food insecurity in the 30 days preceding the survey, including anxiety and uncertainty about access, and insufficient quality and quantity of intake. The households were categorized into two groups—food secure and food insecure (which included the mildly, moderately and severely food-insecure groups). Mild, moderate and severe food insecurity were calculated based on the responses to more severe conditions and/or the frequency of experiencing the conditions following steps described in the manual by Coates *et al.* (2007).

#### Intra-household relationship

Educational discrepancy between a woman and her husband was used as a proxy for potential relationship inequality. Spousal education gap was calculated by deducting the wife’s education from the husband’s education. The couples with an equal educational level were furthermore divided into two subgroups: couples with secondary or higher education and with no spousal education gaps were termed as 'no gap, high education', and, equivalently, couples with no gap and primary or no education were termed as 'no gap, low education'. Thus, this variable had four categories: (1) no gap, low education, (2) wives had higher education than their husbands, (3) husbands had higher education than their wives and (4) no gap, high education.

### Statistical analysis

We first used descriptive analyses to report the characteristics of the study sample as well as experiences of different forms of IPV (controlling behaviour, emotional, physical and sexual violence) among recently delivered women in their lifetime and in the last 12 months. We then used multivariable logistic regression analyses to examine factors associated with different forms of IPV and to assess the association between IPV and maternal CMD, as well as between IPV and breastfeeding practices. Finally, we used structural equation models, considering all the potential variables in our conceptual framework, to examine the complex inter-relationship between these factors and breastfeeding. We presented odds ratios (OR) with 95% confidence intervals (95% CIs) for the multivariable regression models and coefficients in the figure of the path analysis. All models were adjusted for geographical clustering at upazila level using a robust sandwich estimator of the standard errors. Statistical significance was defined as *P* value <0.05. All analysis was done using Stata version 15.1 software (StataCorp, 2019).

## Results

### Characteristics of the study sample

The mean age of mothers was 24 years (range 13–44) (Table 1); >10% of the women were illiterate and only 15% had been educated at high school or a higher level. Two-thirds of the women reported their first marriage before 19 years old. The average of parity was two children, and 22% of women had had a C-section during their last delivery. Nearly half of women lived in food-insecure households.

**Table 1:** Sample characteristics

	*N*	Percent/mean ± SD
Outcomes		
Mental stress scale (range: 0–20)	2000	3.00 (1.00, 7.00)^a^
Common mental disorder ≥7	559	27.95
EIBF	1071	53.55
EBF	1346	67.30
Maternal factors		
Age (years)	2000	24.47 ± 5.51
13–19	404	20.20
20–29	1207	60.35
30–44	389	19.45
Education		
Illiterate	232	11.60
Elementary school	703	35.15
Middle school	758	37.90
High school or higher	307	15.35
Parity	2000	1.98 ± 0.96
1	772	38.60
2	681	34.05
3–4	547	27.35
Early marriage (age <19)	1708	67.15
Caesarean section	445	22.25
Overall autonomy index	1983	15.09 ± 4.12
Low	734	37.01
Medium	750	37.82
High	499	25.16
Household factors		
Household food insecurity	891	44.55
SES (tertile)	655	33.33
Educational inequality		
Education gap (range −11 to 14)	1657	−0.86 ± 3.27
Wife higher educated	786	47.44
Husband higher educated	481	29.03
No gap, low education	252	15.21
No gap, high education	138	8.33
Child factors		
Child age (months)	2000	3.01 ± 1.70
Child female	2000	47.70

a Values are median and interquartile.

The overall autonomy index was 15.1 ± 4.1 (range 2–25) (Table 1). Most women had low levels of asset ownership (13–53%) and purchasing power (40–58%) (Supplementary Table S2). Although a substantial percentage of women decided on family healthcare and movement, 52% of women had supportive attitudes towards wife beating. Regarding educational gaps, there were 47% of couples in which the wife had a higher educational level than their husband. The proportion of spouses who were equally poorly educated was high at 15%, and only 8% of couples were equally highly educated.

### Experiences of IPV

**Figure 2:**
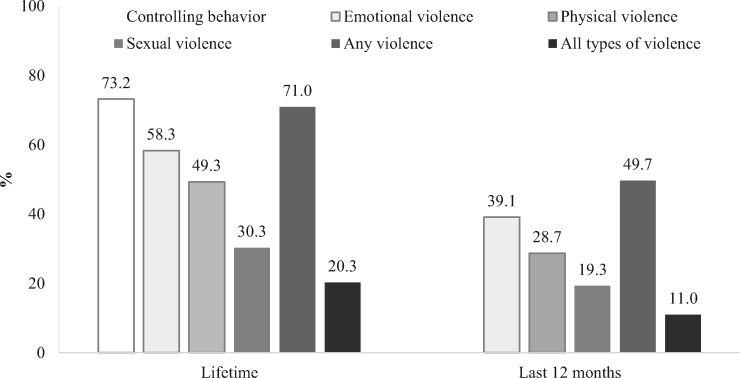
Experiences of IPV in lifetime and in last 12 months

Around three-quarters of women reported one or more controlling behaviour by their husband (Figure 2). The most common acts of controlling behaviour were the husband’s expectation that the wife ask permission before seeking heathcare (60%), insistence on knowing where the wife was at all times (29.2%), and trying to keep the wife from seeing her friends and getting angry if she spoke to another man (20.3%) (Supplementary Table S1).

The lifetime prevalence of domestic violence was high: 58.3% for emotional violence, 49.3% for physical violence and 30.3% for sexual violence; >70% of women had experienced at least one type of IPV and one-fifth of women had experienced all three types of violence in their lifetimes. For the 12 months prior to the survey, half of the women reported experiencing at least one type of IPV and 11% reported experiencing all three types of IPV (Figure 2). Regarding different acts of violence, 37% of women were insulted or made to feel bad about themselves, a quarter were slapped and 18% were physically forced to have sexual intercourse (Supplementary Table S1).

### Determinants of IPV

The four key factors found to be associated with IPV were living in food-insecure households, being of low socio-economic status, having low autonomy and there being inequality in education between the spouses (Table 2). Household food insecurity was significantly associated with higher odds of being faced with controlling behaviour and all types of IPV (OR ranged from 1.6 to 2.3). Women living in the lowest SES households were more likely to experience physical violence and all types of IPV than those in the highest SES households (OR ranged from 1.8 to 2.0). Compared to women with high autonomy, those with low autonomy were 2.5 times (95% CI: 1.6–4.1) more likely to experience controlling behaviour and 1.6 times (95% CI: 1.0–2.5) more likely to experience physical IPV. Inequality in education between husbands and wives increased the likelihood of domestic violence towards women. Couples with higher educated wives showed a significantly higher likelihood of experiencing physical violence than equally highly-educated spouses (OR: 1.88, 95% CI: 1.17, 3.02). Any educational inequality between husbands and wives (OR: 2.29 and 2.25), and an equally low education (OR: 2.79, 95% CI: 1.18–6.55), meant a significantly higher chance of all types of violence compared with equally highly-educated couples (Table 2).

**Table 2:** Factors associated with different types of IPV

	Controlling behaviour	Emotional violence	Physical violence	Sexual violence	All violence
	OR [95% CI]	OR [95% CI]	OR [95% CI]	OR [95% CI]	OR [95% CI]
Maternal age					
13–19	1	1	1	1	1
20–29	1.11 [0.81, 1.53]	0.94 [0.69, 1.27]	0.87 [0.64, 1.18]	0.85 [0.56, 1.30]	0.76 [0.40, 1.43]
30–44	0.9 [0.52, 1.54]	0.82 [0.44, 1.51]	0.73 [0.48, 1.11]	0.93 [0.48, 1.84]	0.64 [0.26, 1.61]
Early married	0.71^**^ [0.56, 0.90]	1.01 [0.63, 1.63]	1.4 [0.94, 2.07]	1.31 [0.86, 2.01]	1.12 [0.57, 2.18]
Parity	1.08 [0.89, 1.32]	1.09 [0.90, 1.33]	1.07 [0.92, 1.25]	0.99 [0.78, 1.24]	1.04 [0.74, 1.44]
Overall autonomy index					
Low	2.53^***^ [1.58, 4.06]	1.11 [0.71, 1.73]	1.59^*^ [1.01, 2.49]	1.43 [0.78, 2.61]	1.73 [0.88, 3.41]
Medium	1.45 [0.99, 2.11]	1.05 [0.76, 1.45]	1.18 [0.80, 1.75]	1.11 [0.72, 1.71]	1.19 [0.70, 2.02]
High	1	1	1	1	1
Household food security					
Secure	1	1	1	1	1
Insecure	1.62^*^ [1.06, 2.48]	2.01^***^ [1.49, 2.70]	2.29^***^ [1.82, 2.88]	1.78^***^ [1.39, 2.27]	2.10^***^ [1.55, 2.83]
Household SES					
Low	1.07 [0.78, 1.46]	1.07 [0.78, 1.47]	1.78^***^ [1.30, 2.43]	1.47 [0.94, 2.30]	2.01^*^ [1.13, 3.56]
Middle	1.09 [0.78, 1.52]	1.16 [0.92, 1.46]	1.26 [0.95, 1.66]	1.04 [0.68, 1.57]	1.47 [0.79, 2.74]
High	1	1	1	1	1
Educational inequality					
Wife more highly educated	0.9 [0.60, 1.34]	1.32 [0.91, 1.89]	1.88^**^ [1.17, 3.02]	1.49 [0.92, 2.42]	2.29^*^ [1.13, 4.64]
Husband more highly educated	0.92 [0.61, 1.41]	1.22 [0.92, 1.63]	1.51 [0.81, 2.82]	1.16 [0.67, 2.01]	2.25^*^ [1.04, 4.85]
No gap, low education	0.76 [0.50, 1.15]	1.34 [0.82, 2.21]	1.8 [0.97, 3.33]	1.31 [0.66, 2.61]	2.79^*^ [1.18, 6.55]
No gap, high education	1	1	1	1	1
Observations	1612	1612	1612	1612	1612

* *P* < 0.05,

** *P* < 0.01,

*** *P* < 0.001.

CI, confident intervals; OR, odds ratio; SES, socio-economic status.

### Association between IPV and maternal CMD

**Figure 3:**
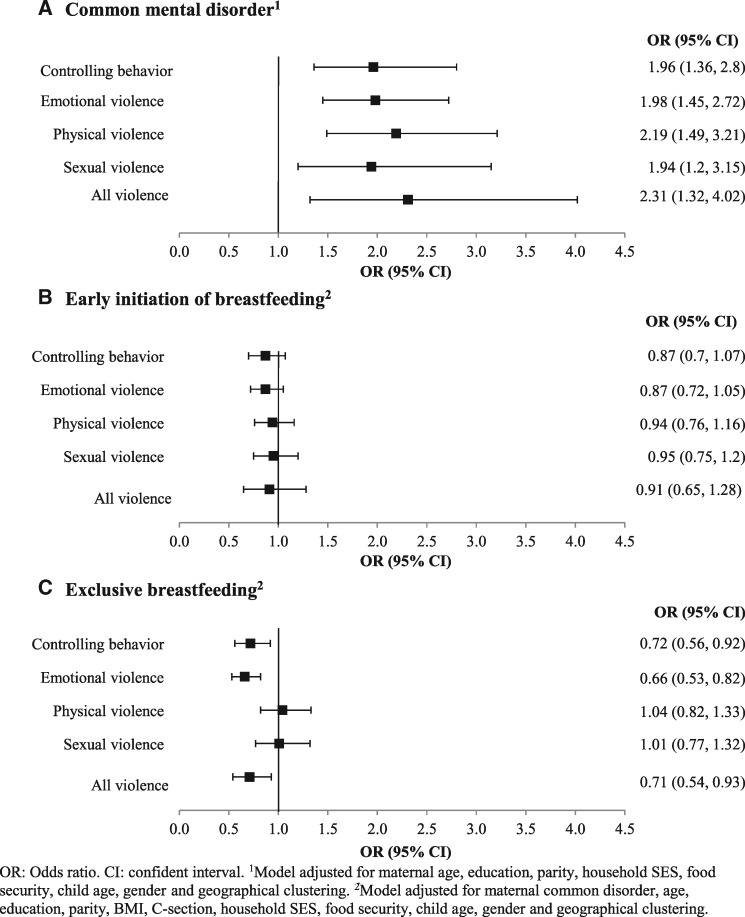
Association between IPV and CMD and breastfeeding practices. (A) CMD^1^. (B) EIBF^2^. (C) EBF^2^. ^1^Model adjusted for maternal age, education, parity, household SES, food security, child age, gender and geographical clustering. ^2^Model adjusted for maternal common disorder, age, education, parity, BMI, C-section, household SES, food security, child age, gender and geographical clustering

The mean score on the CMD scale was 4.7 and the proportion of mothers with CMD was 28% (Table 1). The three most prominent symptoms of CMD were headaches (57%), poor appetite (45%), poor sleep (42%) and difficulty enjoying daily activities (32%) (Supplementary Figure S1). Women who experienced controlling behaviour were more likely to have CMD (OR 1.96; 95% CI 1.36, 2.80) than non-abused women (Figure 3). All types of IPV were significantly associated with CMD (OR 1.9–2.3), and the odds of CMD were highest among women who had experienced all forms of violence (OR 2.31; 95% CI 1.32, 4.02).

### Association between IPV and breastfeeding practices

More than half of the children (54%) had been breastfed within 1 h of birth, and two-thirds of the children under 6 months of age were exclusively breastfed. Mothers exposed to IPV were less likely to initiate breastfeeding early (OR ranged from 0.87 to 0.95); however, the association was not significant at *P* < 0.05. Both controlling behaviour and emotional violence were found to be negatively associated with EBF in the first 6 months of age (Figure 3). Compared with women not exposed to IPV, the odds of EBF were lower among those exposed to controlling behaviour (OR: 0.72, 95% CI: 0.56, 0.92), emotional violence (OR: 0.66, 95% CI: 0.53, 0.82) and all three forms of violence (OR: 0.71, 95% CI: 0.54, 0.93).

### Inter-relationships between determinant factors, IPV, maternal CMD and breastfeeding practices

**Figure 4:**
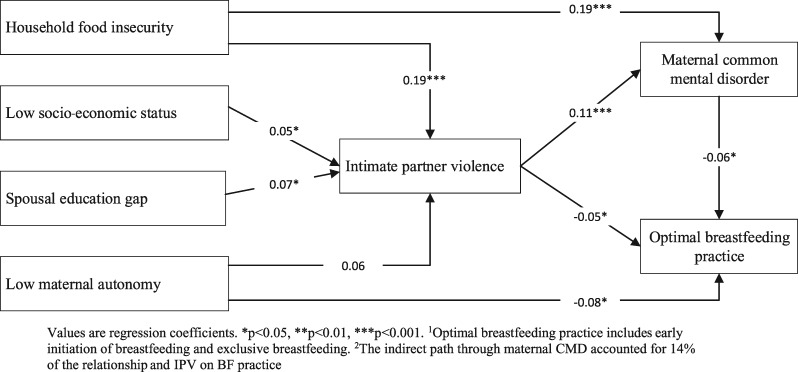
Inter-relationships between determinants factors, IPV, maternal CMD and breastfeeding practices^1^^**,2**^. ^1^Optimal breastfeeding practice includes EIBF and EBF. ^2^The indirect path through maternal CMD accounted for 14% of the relationship between IPV and breastfeeding practice

In the full path model considering all available factors, we found evidence of strong links between potential determinants, IPV, maternal CMD and breastfeeding practice (Figure 4). Household food insecurity, low SES and spousal education gap showed a direct association with experienced IPV (*β* = 0.19, 0.05 and 0.07, respectively, all *P* < 0.05). IPV was directly associated with both maternal CMD (*β* = 0.11, *P* < 0.001) and optimal breastfeeding practice (*β* = −0.05, *P* < 0.05). The indirect path through maternal CMD accounted for 14% of the relationship between IPV and breastfeeding practice. Household food security also indirectly influenced breastfeeding practices, though both IPV and maternal CMD, and low maternal autonomy, had direct associations with breastfeeding practices (*β*= −0.08, *P* < 0.05).

## Discussion

Our study confirms that IPV is pervasive in Bangladesh, affecting more than half of the women in our survey sample during pregnancy and the lactation period. Women with low autonomy, living in poor households, experiencing food insecurity and with unequal education compared with their husbands were more likely to experience IPV. All forms of IPV were positively associated with maternal CMD and negatively associated with EBF practices. The path of influence from IPV to breastfeeding practice through maternal CMD was important, explaining 14% of the relationship.

Although a range of studies have explored the determinants of IPV and its health consequences among married women, our study contributes to the empirical literature by demonstrating the complex relationship between IPV, maternal mental health and breastfeeding practices around the time of pregnancy and lactation—a vulnerable period for women. Due to a mixture of health behaviours and psychological changes in the pregnancy and postnatal periods, women need more care and love including more health check-ups, a better diet, a less heavy workload and more support with household chores and care of other children. Instead of receiving those forms of special care, Bangladeshi women still suffer from very high IPV prevalence in this sensitive period, at nearly 50%. Other research suggests that IPV in this context can contribute to more adverse pregnancy outcomes such as increased risk of preterm birth, low birth weight and intrauterine growth restriction (Donovan *et al.*, 2016; Hill *et al.*, 2016) and poor child feeding practices.

Consistent with other studies (Rahman *et al.*, 2014; VanderEnde *et al.*, 2015; Islam *et al*., 2017a; Caleyachetty *et al*., 2019; Diamond-Smith *et al.* 2019), our analysis indicated that women who live in food-insecure and low SES households were more likely to have experienced IPV. These results imply that attention must be paid to opportunities to mainstream IPV prevention and response through programming in underlying factors such as food security, livelihoods and economic empowerment. We also found evidence of an association between a low level of women’s overall autonomy and a high risk of experiencing controlling behaviour and physical IPV. In the context of the low economic decision-making power of women and widespread attitudes that support wife beating, as reported, women are at high risk of IPV. Our results, therefore, indicate that women’s autonomy, particularly economic decision-making autonomy, and women’s own attitudes towards partner violence may need to be considered as important socio-cultural determinants for reducing the risk of IPV among women in Bangladesh. Regarding relationship inequality between spouses, educational inequality (either the wife or husband had a higher education than their spouse) increased the likelihood of IPV. Couples where both the wife and husband had a low education (less than five years of schooling) had the highest IPV prevalence. Because of the risk of educational discrepancy between spouses, it appears that educational interventions do not have a purely beneficial effect if either men or women are the only ones receiving the education. This circumstance suggests the need for more directed educational interventions for both men and women.

Our study demonstrated that all forms of IPV were associated with maternal CMD, a finding consistent with previous studies in rural Bangladesh and other settings (Dillon *et al.*, 2013; Ziaei *et al.*, 2016). Poor mental health lessens the mother’s capability to take adequate care of her child (Stein *et al.*, 2014), which in turn can have adverse effects on children’s growth (Stewart *et al.*, 2008; Nguyen *et al.*, 2014) and development (Rahman *et al.*, 2008). While results from a previous study in LMICs showed a decreased likelihood of exclusively breastfeeding related to exposure to all forms of IPV (Caleyachetty *et al.*, 2019), our study only found this association with exposure to controlling behaviour and emotional violence. Given the culture of strong influences of the husband and family members on breastfeeding practices, interventions should be designed to engage husbands and the broader community to support mothers to achieve the recommended practices.

Health systems play a crucial role in a multisectoral response to violence against women, including documenting prevalence, primary prevention, emphasizing its health burden and advocating for coordinated action with other sectors (Garcia-Moreno *et al.*, 2015). During pregnancy, antenatal care (ANC) visits have been identified as an important platform for IPV screening and prevention because ANC provides an opportunity to enquire about IPV, and also allows for the possibility of follow-up during ANC with appropriate supportive interventions, such as counselling and empowerment interventions (WHO, 2016). The current WHO recommendation on ANC for a positive pregnancy experience strongly encourages countries to include IPV components at ANC visits when assessing conditions that may be caused or complicated by IPV, to improve clinical diagnosis and subsequent care (WHO, 2016). In addition, providers must be trained to ask questions in the correct way and to respond appropriately to women who disclose violence (WHO, 2013b, 2019). A previous systematic review of the existing literature found that evidence of effective interventions for IPV during the perinatal period is lacking, but home visitation programmes and some multifaceted counselling interventions did produce promising results (OR ranged from 0.47 to 0.92) (Van Parys *et al.*, 2014). Some countries (Spain, India, Lebanon, Brazil and South Africa) have guidelines or protocols to incorporate care for IPV into their healthcare systems, but overall system development and implementation have been slow to progress (Garcia-Moreno *et al.*, 2015).

To address the high burden of IPV, the Bangladesh policy framework has included a number of conventions, policies and acts on violence against women, among them the Suppression of Violence against Women and Children Act in 2000, the Domestic Violence (Prevention and Protection) Act in 2010 and the Domestic Violence (Prevention and Protection) Rules in 2013 (Naved *et al.*, 2017a). In response to the global recommendations, the health sector also included support for IPV in the essential health service package (Gov of Bangladesh, 2016). However, there are substantial gaps in the implementation of laws and policies where the IPV screening guideline was not mandatory in the national strategy for maternal and neonatal health (Gov of Bangladesh, 2009). Given that health platforms play a critical role in reaching pregnant women and their families, IPV screening and support services, including couple counselling sessions, should be included as part of routine antenatal care to ensure that all pregnant women receive appropriate support. Some governments and professional organizations recommend screening all women for IPV rather than asking only women with symptoms (WHO, 2013b), and it is imperative that this be prioritized in Bangladesh, given the very high IPV burden and the range of poor health outcomes that are associated with IPV.

Previous studies in Bangladesh have shown positive effects of breastfeeding counselling on mitigation of the negative impact of IPV on the duration of EBF (Frith *et al.*, 2017). Combining cash transfers with group-based nutrition behaviour change communication had an impact not only on nutrition behaviours (Hoddinott *et al.*, 2018) but also on IPV (Roy *et al.*, 2017) in Bangladesh. This suggests that combined intervention may have positive impacts on IPV and on nutrition behaviours. Given the effectiveness of large-scale maternal nutrition (Nguyen *et al.*, 2017a) and child feeding programmes (Menon *et al.*, 2016) that include interpersonal counselling and community mobilization, adding an IPV component to the counselling content and involving men in the counselling sessions can bring additional benefits to reduce the prevalence of IPV, improving maternal mental health and amplifying effects on child breastfeeding practices. As noted above, mandating and supporting the integration of IPV screening and support as part of routine care during pregnancy could go a long way in recognizing the challenge and creating supportive structures within the health system.

Our study has some limitations. To collect information on mothers and on feeding for children born to those mothers, we only included women with living children <6 months, thus excluding women who died or whose babies died. We acknowledge that those women may be the most vulnerable to IPV and its consequences. The assessment of breastfeeding practices was based on mothers’ reports of all foods and liquids given to children in the first few days after birth (for EIBF) and in the 24 h prior to the survey (for EBF), which may be subject to recall bias. Given that all mothers reported on feeding practices that were ongoing or within the last six months, we believe, however, that mothers would have had good recall. Data for CMD were also collected based on maternal recall of their symptoms of mental disorders in the last four weeks. Maternal recall has its limitations and CMD may influence the recall, either through under- or over-reporting of information. Due to the study’s cross-sectional design, we are not able to establish causal relationships between variables, but we can infer associations. Finally, our data were collected from rural areas, thus they have limited generalizability at the national level.

Despite these limitations, the study findings have important implications for policy and practice in Bangladesh based on insights into the complex relationship between factors influencing IPV, as well as the adverse consequences of IPV on maternal mental health and child feeding practices. Given the high IPV prevalence in Bangladesh, integrating actions both to reduce IPV and to mitigate the impacts of IPV into existing prenatal and postnatal care services, can serve as multiple duty actions to simultaneously address the high prevalence of IPV, poor maternal mental health and suboptimal breastfeeding practices. Several options are available to policymakers, at least some of which have been tested in rural Bangladesh, but further action is urgently needed given the high prevalence of IPV as well as the huge burden that exposure to IPV places on the lives of women and their children in this context.

## Supplementary data

Supplementary data are available at *Health Policy and Planning* online.

## Supplementary Material

czaa106_Supplementary_Files
