# Supplementary material for: Intimate partner violence is associated with poorer maternal mental health and breastfeeding practices in Bangladesh
Source: Health Policy Plan. 2020 Nov 9;35(Suppl 1):i19–29. doi: 10.1093/heapol/czaa106 (PMC7649668; doi:10.1093/heapol/czaa106)
Supplement: czaa106_Supplementary_Files [file czaa106_supplementary_files.docx]

Supplementary Table 1. Prevalence of different acts of violence

|  | **Lifetime** | **In the last 12 months** |
| --- | --- | --- |
|  | **%** | **%** |
| **Controlling behavior** |  |  |
| Tries to keep you from seeing your friends | 21.3 |  |
| Tries to restrict you from contacting your family of birth | 19.8 |  |
| Insists on knowing where you are at all times | 29.2 |  |
| Ignores you and treats you indifferently | 9.9 |  |
| Gets angry if you speak to another man | 20.3 |  |
| Is often suspicious that you are unfaithful | 9.3 |  |
| Expects you to ask his permission before seeking health care for yourself | 60.2 |  |
| *At least one controlling behavior* | 73.2 |  |
| **Emotional violence** |  |  |
| Insulted you or made you feel bad about yourself | 56.2 | 37.3 |
| Belittled or humiliated you in front of other people | 26.1 | 17.3 |
| Done things to scare or intimidate you on purpose | 15.6 | 10.1 |
| Threatened to hurt you or someone you care about | 4.1 | 2.7 |
| *Any emotional violence* | 58.3 | 39.1 |
| **Physical violence** |  |  |
| Push you, shake you, or throw something at you | 18.6 | 11.0 |
| Slap you | 46.1 | 24.8 |
| Twist your arm or pull your hair | 11.6 | 6.4 |
| Punch you with his fist | 12.6 | 7.0 |
| Kick you, drag you or beat you up | 11.8 | 6.4 |
| Try to choke you or burn you on purpose | 1.9 | 1.1 |
| Threaten or attack you with a knife, gun, or any other weapon | 1.7 | 1.2 |
| *Any physical violence* | 49.3 | 28.7 |
| **Sexual violence** |  |  |
| Physically force you to have sexual intercourse when you did not want to | 27.7 | 17.8 |
| Have sexual intercourse you did not want to because you were afraid of what your partner or any other partner might do | 12.6 | 7.3 |
| Forced you to do something sexual that you found degrading or humiliating | 9.8 | 6.0 |
| *Any sexual violence* | 30.3 | 19.3 |
| **Any violence** | 71.0 | 49.7 |
| **All types of violence** | 20.3 | 11.0 |

Supplementary Figure 1. Women’s symptoms of common mental disorder

Supplementary Table 2. Women’s autonomy indicators

|  | **Percent/ Mean ± SD** |
| --- | --- |
| **Economic power decision index** |  |
| Ownership of land | 13.95 |
| Ownership of house where usually live | 22.35 |
| Ownership of other house | 12.60 |
| Ownership of big animals like cows, horses, donkey | 28.55 |
| Ownership of small animals like hens, ducks | 52.60 |
| Ownership of gold jewelry | 87.70 |
| Decide to buy food like rice, vegetable | 40.40 |
| Decide to buy animal source foods | 40.55 |
| Decide to buy cooking oil | 47.40 |
| Decide to buy medicine for yourself | 56.25 |
| Decide to buy medicine for children | 58.35 |
| Decide if have to work to earn money | 51.30 |
| **Familial health care and family planning decision index** |  |
| Decide what food is prepared every day | 70.05 |
| Decide use of family planning methods | 62.20 |
| Decide to eat nutritious food during pregnancy | 81.60 |
| Decide to take supplemental tablets during pregnancy | 89.40 |
| Decide to take rest every day for a certain time during pregnancy | 94.55 |
| Decide whether or not you breastfeed the child and when to wean the child | 96.70 |
| Decide what and how to feed the infant in his first year of life | 95.45 |
| **Index for freedom of movement** |  |
| Decide visiting other family members, friends or relatives | 57.30 |
| Decide seeing a doctor or visiting a dispensary when you are pregnant | 62.20 |
| **Index of women’s attitudes toward gender:** women agree with the declarations**:** |  |
| In a household, the man should take the important decisions^1^ | 44.02 |
| If the woman works outside home, her husband or partner should help her with the daily housework. | 93.35 |
| A husband should not let his wife work outside home, even if she would like to do it^1^ | 46.54 |
| A woman has the right to express her opinion if she does not agree with what the husband or partner says. | 86.43 |
| A woman must accept that her husband or partner beats her in order to keep the family together^1^ | 52.35 |
| It is better to send a son to school than a daughter^1^ | 88.04 |
| **Overall autonomy index (range 2 – 25)** | 15.09 ± 4.12 |

^1^ Reverse code to calculate overall autonomy index
